# Quantitative analysis of the caloric restriction versus isocaloric diets models based on macronutrients composition: impacts on body weight regulation, anthropometric, and bioimpedance parameters in women with obesity

**DOI:** 10.3389/fnut.2024.1493954

**Published:** 2024-12-12

**Authors:** Denisa Pescari, Monica Simina Mihuta, Andreea Bena, Dana Stoian

**Affiliations:** ^1^Department of Doctoral Studies, Victor Babeș University of Medicine and Pharmacy, Timisoara, Romania; ^2^Center for Molecular Research in Nephrology and Vascular Disease, Victor Babeș University of Medicine and Pharmacy, Timisoara, Romania; ^3^Discipline of Endocrinology, Second Department of Internal Medicine, Victor Babeș University of Medicine and Pharmacy Timisoara, Timisoara, Romania

**Keywords:** obesity, overweight, macronutrient distribution, body composition changes, adipose tissue, bioimpedance metrics

## Abstract

**Introduction:**

Obesity is a growing public health issue, especially among young adults, with long-term management strategies still under debate. This prospective study compares the effects of caloric restriction and isocaloric diets with different macronutrient distributions on body composition and anthropometric parameters in obese women during a 12-week weight loss program, aiming to identify the most effective dietary strategies for managing obesity-related health outcomes.

**Methods:**

A certified clinical nutritionist assigned specific diets over a 12-week period to 150 participants, distributed as follows: hypocaloric diets—low-energy diet (LED, 31 subjects) and very low-energy diet (VLED, 13 subjects); isocaloric diets with macronutrient distribution—low-carbohydrate diet (LCD, 48 subjects), ketogenic diet (KD, 23 subjects), and high-protein diet (HPD, 24 subjects); and isocaloric diet without macronutrient distribution—time-restricted eating (TRE, 11 subjects). Participants were dynamically monitored using anthropometric parameters: body mass index (BMI), waist circumference (WC), waist to hip ratio (WHR) and bioelectrical impedance analysis (BIA) using the TANITA Body Composition Analyzer BC-418 MA III (T5896, Tokyo, Japan) at three key intervals—baseline, 6 weeks, and 12 weeks. The following parameters were evaluated: body weight, basal metabolic rate (BMR), percentage of total body fat, trunk fat, muscle mass, fat-free mass, and hydration status.

**Results:**

All diets led to weight loss, but differences emerged over time. The TRE model resulted in significantly less weight loss compared to LED at the final follow-up (6.30 kg, *p <* 0.001), similar to the VLED (4.69 kg, *p <* 0.001). Isocaloric diets with varied macronutrient distributions showed significant weight loss compared to LED (*p <* 0.001). The KD reduced waist circumference at both 6 and 12 weeks (−4.08 cm, *p <* 0.001), while significant differences in waist-to-hip ratio reduction were observed across diet groups at 12 weeks (*p* = 0.01). Post-hoc analysis revealed significant fat mass differences at 12 weeks, with HPD outperforming IF (*p* = 0.01) and VLED (*p* = 0.003). LCD reduced trunk fat at 6 weeks (−2.36%, *p* = 0.001) and 12 weeks (−3.79%, *p <* 0.001). HPD increased muscle mass at 12 weeks (2.95%, *p* = 0.001), while VLED decreased it (−2.02%, *p* = 0.031). TRE showed a smaller BMR reduction at 12 weeks compared to LED.

**Conclusion:**

This study highlights the superior long-term benefits of isocaloric diets with macronutrients distribution over calorie-restrictive diets in optimizing weight, BMI, body composition, and central adiposity.

## 1 Introduction

Overweight and obesity, with their multifaceted nature and numerous related complications, are widely acknowledged as significant public health concerns. Environmental factors, the most known such as dietary habits and physical inactivity play a major role in the increasing incidence of excess weight, despite the well-established contributions of genetic and epigenetic factors (1). According to The Lancet, over 1 billion people worldwide have been projected to be affected by obesity since 2022 (2). Moreover, the global prevalence of overweight individuals has doubled since 1990, reflecting a significant and concerning trend. This increase is particularly alarming in children aged 5–19, where the incidence has surged fourfold over the same period (3). Obesity has been associated not only with cardiovascular diseases and insulin resistance but also with a broader increase in overall morbidity and mortality rates (4). Consequently, a high value of BMI is estimated to be a major contributing factor to approximately 60% of deaths worldwide, underscoring the severe health risks associated with excess weight (5). Global projections indicate that by 2030, obesity will affect 1 billion adults worldwide, transcending distinctions of gender, geographic regions, and differences between rural and urban living environments (6, 7). This alarming statistic highlights the critical impact of excess weight on global health, emphasizing the urgent need for targeted interventions and comprehensive public health strategies to address and mitigate the factors contributing to this widespread issue.

Although BMI is widely regarded as the most common tool for assessing an adult’s nutritional status and estimating generalized adipose tissue (8, 9), it has several significant limitations. One of the primary drawbacks is its inability to differentiate between the segmental distribution of adipose tissue, which can be crucial for assessing health risks (10–13). Furthermore, BMI may not be a reliable indicator during the monitoring of weight loss dynamics in individuals with obesity, as it does not reflect changes in body composition, such as muscle gain or fat loss, which are important for evaluating progress in weight management programs. In addition to BMI, there are other girth measurements that can assess adipose tissue distribution both at baseline and dynamically in adults with excess weight. Among the most well-known are WC and WHR (14). WC is recognized as a valuable tool, closely correlated with visceral or abdominal adipose tissue, thereby serving as a predictor of potential obesity-related complications (15, 16). Alongside WC, WHR is an equally important anthropometric marker for evaluating the increase in insulin resistance (17). These parameters can be easily and effectively monitored over time during the weight loss process, offering valuable insights into the progression and potential health outcomes. Decreasing visceral fat and body size, particularly WC, in individuals with obesity is essential for enhancing both physical and mental health and also for sustaining motivation throughout the treatment process (14). However, they do not provide comprehensive information about body composition, such as the relative proportions of adipose, muscle, and other tissues. In this context, for a more in-depth evaluation of an adult’s nutritional status, as well as for dynamic monitoring during a weight loss program, body composition analysis through BIA is extremely valuable.

With the advancement of new technologies, it is now possible to determine various body masses and their distribution both in kilograms and as percentages. Over time, several devices have been validated for this purpose: dual-energy X-ray absorptiometry (DXA), computed tomography (CT), and magnetic resonance imaging (MRI). However, the most commonly used method today is BIA, a technique that is easy to use, non-invasive, and relatively inexpensive (18–21). These are just a few of the advantages that characterize BIA, making it a practical tool widely recognized in both research and clinical nutritional practice.

Long-term management of individuals with excess weight presents several challenges, but the most significant is the relationship between the body’s energy conservation mechanisms and heightened sensitivity to appetite-stimulating factors, which complicates the prevention of weight regain after initial weight loss (22, 23). Over the course of human evolution, the survival instinct has likely driven the body to prioritize the accumulation and preservation of fat stores. This adaptive mechanism would have been crucial in times of food scarcity, ensuring energy reserves were available during periods of famine. As a result, the human body has evolved to efficiently store fat, a trait that, while advantageous in the past, now contributes to the global rise in obesity in modern environments where food is abundant (24, 25). Multiple strategies are currently employed for managing weight in individuals with obesity. Various weight loss interventions, including dietary modifications, physical activity programs, behavioral therapy, pharmacotherapy, and bariatric surgery, have been developed to combat obesity (26). These interventions aim not only to reduce BMI but also to mitigate the associated health risks and improve overall quality of life (22, 24). Therefore, calorie restriction and increased physical activity are commonly recommended as the initial steps in most weight loss programs (27). These strategies aim to create a calorie deficit, which is essential for reducing body weight and improving overall health outcomes. A general definition of “diet” refers to the total amount of energy and nutrients derived from the foods and beverages that individuals regularly consume (28). Typically, the most recommended nutritional approach involves an increase in complex or unprocessed carbohydrates and a reduction in fat intake, with the goal of decreasing overall energy intake while boosting energy expenditure through physical activity (29, 30). Nevertheless, lifestyle modification programs often fall short of achieving success, especially in individuals with severe obesity (30).

Diets have been shown to play a critical role in influencing weight loss and managing obesity, particularly among women, as their metabolic and hormonal responses to dietary interventions may differ from men (31). Studies suggest that women are more likely to engage in weight-loss diets and experience a higher prevalence of dieting behavior compared to men, driven by societal pressures and health-related concerns (32). Caloric restriction, a common feature in most weight-loss diets, can lead to significant reductions in body weight and adipose tissue in women, although the outcomes are influenced by factors such as age, baseline weight, and metabolic rate (33). Additionally, some research has indicated that women may experience a slower rate of weight loss compared to men when following similar dietary regimens, possibly due to differences in adipose tissue distribution, resting energy expenditure, and hormonal fluctuations, such as those associated with estrogen and progesterone (34). Furthermore, long-term adherence to specific dietary patterns, such as low-carbohydrate or low-fat diets, has been found to impact weight maintenance differently in women, with varying degrees of success depending on individual metabolic profiles and lifestyle factors (35).

Low-energy diets (LEDs) are typically defined as providing 800–1,200 kcal per day, although some definitions extend this range to include diets that provide between 800 and 1800 kcal per day (36, 37). These types of diets are commonly employed in the management of obesity due to their effectiveness in promoting rapid weight loss (38, 39). Compared to LEDs, very low-calorie diets (VLEDs) are defined by an intake of only 400–800 kcal per day (36). Most VLEDs rely on commercially prepared semisolid or liquid foods designed to induce rapid weight loss (28). Although VLEDs are commonly used in nutritional management for severe obesity, typically for periods of up to 12 weeks, the maximum safe duration for maintaining this type of diet therapy remains unclear (28). Furthermore, inadequate medical supervision and a reduction in muscle mass due to insufficient protein intake can lead to severe consequences, including death (40). This risk is associated with the different macronutrient proportions in VLEDs, where the protein intake is higher (ranging from 1.2 to 1.5 g/kg/day) compared to other diets (41). Among individuals with obesity, significant caloric restriction has been shown to be effective for achieving substantial weight loss, with greater long-term success (42). However, it has been observed that VLEDs do not result in greater long-term weight loss compared to LEDs (37). Additionally, the clinical applicability of this dietary approach is limited for athletes and individuals without excess weight (28).

In recent years, the growing popularity of low-carbohydrate diets (LCDs) has sparked a significant increase in research examining their effects on a wide range of metabolic conditions and non-disease states (43, 44). However, there is still no universally established definition for the total carbohydrate content in such diets (28). The Acceptable Macronutrient Distribution Range (AMDR) suggests that for adults, carbohydrates should constitute 45 to 65% of the total macronutrient intake (45). Therefore, any diet providing <45% of total daily energy intake from carbohydrates can be classified as a LCD. Some definitions not aligned with AMDR guidelines consider a more stringent reduction, with carbohydrate intake below 40% (46, 47). In addition to percentage-based definitions, the absolute number of grams of carbohydrate intake has also been used to define an LCD. Specifically, a diet providing <200 g of carbohydrates per day is considered a LCD, while others classify non-ketogenic LCDs as those providing 50–150 g of carbohydrates per day (46, 48). It is important to note that a carbohydrate intake ranging between 50 and 55% of total energy has been associated with a reduced risk of mortality in modern settings (45). Additionally, it has been suggested that the quality of ingested carbohydrates may have a more significant impact on health outcomes than merely focusing on the total carbohydrate intake (49).

The KD, commonly known for its low carbohydrate and high fat intake, is designed to promote weight loss (28). Numerous studies have demonstrated that ketogenic diets can lead to rapid weight loss (50–52). While initial weight loss and reduction in adipose tissue occur as a result of strict adherence to this diet, these effects tend to diminish over time, becoming comparable to other weight loss dietary approaches after 1 year (53, 54). This type of diet is similar to LCD, with the key difference being that the total daily carbohydrate intake in a ketogenic diet is limited to a maximum of 50 g of carbohydrates or 10% of total macronutrient intake (48, 54). Given the macronutrient distribution principle of this diet (55–60% fats, 30–35% protein, and only 5–10% carbohydrates), it induces ketosis, a metabolic state in which the body primarily utilizes fat, rather than carbohydrates, as its main energy source (55). Currently, four subtypes of the ketogenic diet are recognized: the modified Atkins diet, the low glycemic index diet, the classic keto diet with long-chain triglycerides, and the keto diet with medium-chain triglycerides (55). Although the ketogenic diet offers benefits such as weight loss and a reduced risk of developing conditions like type 2 diabetes, cardiovascular diseases, and certain cancers (56), it is also associated with several short-term adverse effects. The long-term effects, however, remain unclear due to limited information (54, 57, 58). The short-term adverse effects, commonly referred to as the “keto flu,” include symptoms such as fatigue, headaches, insomnia, nausea, vomiting, and constipation (55). While these short-term effects can be managed, the long-term effects are more concerning and may lead to conditions such as hypoproteinemia, hypercalciuria, kidney stones, and vitamin deficiencies (59, 60).

Protein is the most crucial macronutrient for promoting positive changes in body composition (61). For active individuals, protein intakes ranging from 1.2 to 2.0 grams per kilogram of body weight per day (g/kg/d) are recommended (59–68). In contrast, the US recommended daily allowance (RDA) for protein is set at 0.8 g/kg/d (69). However, consuming amounts above the RDA may be considered a “high” protein intake (70). A moderate energy restriction of 500–750 kcal/day, combined with a protein intake exceeding 22% of total macronutrients, has been shown to confer benefits such as increased insulin sensitivity (71–73) and improved preservation of lean body mass during weight loss (74–76). Additionally, it has been observed that overweight women following a calorie-restricted diet with a 750 kcal deficit and a protein intake of 30% (1.4 g/kg/d) over a 12-week period experienced a smaller reduction in lean body mass compared to those on isocaloric diets with an optimal protein intake of 0.8 g/kg/day (77). Similar results have been observed in men (78). However, the impact of these types of diets on adipose tissue mass is less well-studied (79).

Intermittent fasting (IF) has gained increasing popularity in recent years due to its ability to promote significant weight loss and provide metabolic protection (80, 81). IF emphasizes the timing of food consumption rather than the total quantity consumed (82). The broad category of IF includes several subtypes: zero-calorie alternate-day fasting (zero-calorie ADF), modified alternate-day fasting (MADF), the 5:2 diet, and TRE (82–84). TRE is considered the most popular form (85). This approach involves limiting food intake, without calorie restriction or monitoring, to a specific time window of 4–10 h, followed by fasting for the remainder of the day (80). Thus, it has been observed that in individuals with obesity who adopt this nutritional strategy, caloric intake can be reduced by up to 550 kcal/day, and weight loss may range between 5 and 10% (86–89). This weight reduction is primarily attributed to a decrease in adipose tissue mass compared to muscle mass (80). Additionally, TRE can lead to a reduction in abdominal and visceral adipose tissue when the overall weight loss is clinically significant (90, 91).

Therefore, the aim of this prospective observational study is to conduct a detailed comparative quantitative analysis of the effects on body composition and anthropometric indices in obese women undergoing a 12-week monitored weight loss program. The study will compare diets based on caloric restriction with isocaloric diets that incorporate variations in macronutrient distribution. The study focuses on evaluating how different dietary patterns for weight loss influence critical parameters such as adipose tissue distribution, muscle mass using BIA and general anthropometric measurements. By investigating these relationships, the research aims to identify the most effective dietary strategies for optimizing body composition and managing obesity-related health outcomes in this population. This analysis is intended to contribute valuable insights to the field of obesity treatment and prevention, with a particular focus on the role of dietary interventions.

## 2 Materials and methods

The prospective observational study was conducted over a period of approximately 3 years, from September 2021 to March 2024, at our endocrinology and nutrition unit. The study cohort comprised 150 females with varying degrees of overweight and obesity, with a mean age of 37.67 ± 13.27, who were willing to assess their eating habits and undergo a comprehensive evaluation of their nutritional status with the aim of lifestyle modification and the development of a dietary program for weight reduction to achieve nutritional goals. Each participant underwent clinical and nutritional assessments at three key points during the study—initial assessment, at 6 weeks, and at the final assessment at 12 weeks—conducted by a certified clinical nutritionist.

Informed consent was obtained from all participants. The study was conducted in accordance with the ethical standards of the Helsinki Declaration and received approval from the Scientific Research Ethics Committee (CECS) of the Victor Babeș University of Medicine and Pharmacy Timișoara (no. 69/03.10.2022).

### 2.1 Patient inclusion and exclusion criteria

Inclusion criteria included female patients aged over 18 years with a BMI >25 kg/m^2^ who voluntarily sought nutritional counseling services to implement a weight loss dietary plan aimed at achieving an ideal weight. The cohort comprised women from both urban and rural areas, with diverse educational backgrounds, varying marital statuses (single, married, divorced, or widowed), and different employment statuses (housewife, student, employed, or retired), including both menopausal and non-menopausal women. Only those patients who agreed to complete the entire evaluation process and provided signed informed consent were included in the final analysis. Additionally, in the final analysis, only women who fully adhered to the established dietary program and attended all three scheduled medical visits were included.

Exclusion criteria: patients under 18 years of age, pregnant or breastfeeding women, those consuming dietary supplements or anti-obesity medications (92), patients with diabetes mellitus on oral antidiabetic drugs with a risk of hypoglycemia (e.g., sulfonylureas) (93) or those undergoing insulin therapy, women who had followed any diet therapy within the past 6 months or prior bariatric surgery, patients with a history of acute pancreatitis, renal or liver diseases, including chronic kidney disease, liver failure, those diagnosed with porphyria, patients who did not agree to sign the informed consent, women who did not adhere to both the prescribed dietary program and regular medical visits, patients with documented psychiatric disorders, and patients with obesity due to specific etiologies, such as genetic conditions (Prader-Willi syndrome), iatrogenic causes (insulin therapy, glucocorticoids, antipsychotics) orendocrinologic disorders (Cushing’s syndrome, hypothyroidism, hypogonadism) were excluded from the study (94, 95). Women with daily alcohol consumption were also excluded from the study.

### 2.2 Patient complete evaluation

Before any procedures were performed, participants were thoroughly informed about the study details, as well as the necessary clinical and paraclinical examinations. Each patient was provided with an informed consent form, which they were required to review and sign. The primary non-invasive method employed in our investigation was bioimpedance measurement, aimed at estimating segmental body composition. During the initial consultation, the anamnesis focused on demographic factors, personal medical history, behavioral factors, and laboratory analyses from the past 6 months. Based on these variables, various population subgroups were formed.

Demographic factors of interest included:

- *Educational level* categorized by years of education (<9 years, 10–12 years, 12–18 years, and > 18 years) (96);
- *Marital status*: single, married, divorced, or widowed;
- *Employment status*: housewife, student, employed, or retired.

Behavioral and lifestyle factors considered were:

- *Smoking status* was classified as positive if the participant smoked at least one cigarette daily for more than 1 year;
- *Physical activity level*: a threshold of a minimum of 150 min per week or at least 30 min per day (activity level above active plus basal) to avoid being classified as sedentary;
- *Sleep duration*: a nightly duration of <7 h was classified as sleep deprivation (97);
- *Alcohol consumption*: participants were asked to self-report their alcohol intake by specifying the number of alcohol units consumed, with one unit defined as equivalent to 10 mL of pure ethanol. Two units equaled a pint or can of beer, one unit corresponded to a 25 mL shot of hard liquor, and one unit was equivalent to a standard 175 mL glass of wine. Participants consuming more than two units of alcohol daily were classified as “alcoholic,” while those who never consumed alcohol were classified as “non-alcoholic” (98).

Personal medical history: Biological data collected at baseline, not older than 6 months, included: fasting glucose (mg/dl), lipid profile: total cholesterol (TC) (mg/dl), low-density lipoprotein (LDL-c) (mg/dl), high-density lipoprotein (HDL-c) (mg/dl), triglycerides (TG) (mg/dl), non-HDLc (mg/dl), uric acid (mg/dl), glycated hemoglobin (HbA1c) (%), thyroid-stimulating hormone (TSH), free thyroxine (FT4), homeostasis model assessment-estimated insulin resistance (HOMA-IR), and 25-OH-Vitamin D (ng/mL). Based on abnormalities in these data, patients were categorized into subgroups including hyperuricemia, lipid profile alterations, vitamin D deficiency, type 2 diabetes, and insulin resistance. Additionally, the menopausal status of each participant was documented.

Nutritional status: was determined by calculating BMI, a commonly used and cost-effective anthropometric parameter, for each participant in the study. BMI was calculated using the formula: BMI = weight (in kg)/height^2^ (in m^2^) (99, 100).

- *Height measurement*: The height of each patient was measured using a mounted, calibrated stadiometer. Each participant was instructed to stand upright on the platform, without wearing shoes, to ensure an accurate assessment of vertical posture.
- *Body weight measurement*: The body weight of each participant at the time of presentation was measured using a metrologically certified mechanical scale, with a maximum capacity of 180 kg. The basic procedure was explained to each subject, who involved maintaining an upright posture on the device while wearing minimal clothing.
- *Waist circumference* was measured at the midpoint between the lower margin of the last palpable rib and the top of the iliac crest, while hip circumference was measured at the widest portion of the buttocks, using a non-elastic measuring tape positioned parallel to the floor. All measurements were conducted by a physician from the research team, ensuring accuracy and reliability in adherence to established anthropometric standards. Body weight was assessed using a calibrated digital scale with participants in a fasting state, barefoot, and wearing light clothing. Measurements were taken at a consistent time of day to minimize diurnal variations, and participants were advised to avoid intense physical activity or hydration changes prior to the assessment to ensure standardized conditions.

### 2.3 Assessment of bioimpedance parameters using bioelectrical impedance body analysis

The Tanita Body Composition Analyzer BC-418 MA III (T5896, Tokyo, Japan) was the segmental body composition analyzer utilized in this study. This device provides validated body measurements comparable to those obtained through DXA, using segmental analysis (101, 102). Several studies have validated that, in clinical environments, the Tanita Body Fat Monitor provides measurements within +/− 5% of the accuracy achieved by the institutional standard for body composition analysis, DXA (103–105). The analyzer operates by measuring impedance through a low-level electrical current (106). Tanita highlights this method as accessible and convenient for accurately quantifying body composition (107). The Tanita body fat monitoring series consistently delivers results with a variation of +/− 1% when used under stable conditions (107). Each participant was instructed to maintain an upright, still position during the procedure, holding the device’s handles to ensure proper connection with the eight electrodes, two for each limb. During the BIA measurements, specific methodological steps were followed to ensure accuracy and reliability. All participants were instructed to remove metallic elements such as jewelry, watches, and belts prior to the procedure, as these could interfere with the bioelectrical signal. Additionally, all measurements were conducted without footwear, with the participants standing barefoot on the device to ensure proper electrode contact and accurate readings. These protocols were implemented consistently across all measurements to minimize variability and maintain the integrity of the data. These measurements were conducted three times throughout the study: at baseline, at 6 weeks, and at 12 weeks (or the final assessment). The parameters assessed using this technique were categorized as follows: fat mass (%), muscle tissue (%), fat-free mass or lean mass (FFM) (kg), trunk adipose mass (%), total body water or hydration status (%) and BMR. The instrument model’s operating system recorded the following personal information: identification data, gender, birth date, and height (cm). After inputting the personal data for each patient, the system automatically calculated the BMR values. The decision not to use raw BIA data for estimating fat and fat-free mass through different equations was based on several methodological considerations. The measurement protocol followed a standing position, as it aligns with the specific configuration of the device used and ensures consistency across participants. Electrodes were placed according to the manufacturer’s guidelines to maintain accuracy and reproducibility. Pre-assessment conditions, such as fasting, hydration status, and physical activity, were not strictly controlled, which may influence the reliability of raw BIA data for equation-based estimations. Additionally, repeated measurements were not subjected to a standardized rest period, further limiting the consistency required for using raw impedance data in predictive equations. These factors justified the reliance on the device’s internal algorithms for body composition analysis, ensuring a standardized approach throughout the study.

### 2.4 Weight management intervention program

Following the initial medical and nutritional consultation, each participant was assigned a dietary regimen by a certified clinical nutritionist, to be followed for a period of 12 weeks. Caloric and macronutrient calculations were strictly individualized based on the initial nutritional status assessment, adhering objectively to the nutritional principles for each dietary category outlined below. The dietary programs were implemented over the entire 12-week period, with meals distributed as 3 main meals and 2 snacks per day. Food selection was guided by the precise reading of each nutritional label, in accordance with national regulations (108). Additionally, beyond the implementation of individualized diet therapy, general nutritional recommendations associated with a healthy lifestyle were provided to each participant. These included the consumption of at least 2.5 liters of non-caloric fluids/water per day, drinking coffee without added sugars or milk, and excluding any foods not included in the prescribed diet plans during the study period. To ensure adherence to the study protocol, daily meal monitoring was conducted via an online platform, and nutritional follow-up visits, including bioimpedance body composition analysis, were scheduled at 6 and 12 weeks. The dietary interventions were selected collaboratively by the physician, nutritionist, and patient after the initial nutritional assessment and included the following categories: caloric restrictive diets or hypocaloric (LED and VLED), isocaloric diets with macronutrient distribution (LCD, KD and HPD) and isocaloric without macronutrient distribution (TRE). The final caloric intake was determined based on the basal metabolic rate (measured by bioelectrical impedance at baseline) multiplied by the PAL (mid-point of the moderately active lifestyle) specific to each participant (109, 110). Except for LED and VLED, the remaining diet therapy categories adhered to an isocaloric model, without any restrictions on energy requirements, with only the macronutrient distribution being tailored to the specific diet category and adjusted according to the individual caloric needs of each participant as follows:

The LED was a balanced diet that included a caloric deficit ranged between 500 and 750 kcal for each participant; the final metabolic expenditure ranged between 800 and 1,200 kcal (36), fully individualized for each participant. The macronutrient distribution consisted of 45–55% carbohydrates, 15–25% proteins, and 25–35% fat (111, 112).

The VLED differed from the LED by providing a lower caloric intake, ranging between 400 and 800 kcal/day (36). The foods required in this diet were primarily freshly prepared liquids and semisolids; no commercially available meal replacements or shake-type products were used. The minimum protein intake was set at 40 g/day, and to counteract constipation, a frequently associated adverse effect, fiber intake was supplemented by an additional 10 g (113).

The LCD focused on a healthy composition, reducing refined carbohydrates (favoring whole grains, fruits, and non-starchy vegetables), increasing plant-derived proteins, and incorporating healthy fats (114). The diets were isocaloric, with adjustments made solely to the macronutrient balance. In this category, carbohydrates accounted for 11–25% of the total energy intake, no more than 130 g/day, with the reduced carbohydrate content compensated by an increase in fats, while protein intake remained unchanged (54, 115).

KD, compared to LCD, was characterized by a carbohydrate intake of less than or equal to 10% (a maximum of 50 g of carbohydrates per day) (54), which was primarily replaced by healthy fats and proteins, maintaining an isocaloric diet. The classic ketogenic diet was followed, adhering to the traditional 4:1 ratio of fat to protein and carbohydrates (approximately 60% fat, 30% protein, with macronutrient distribution varying between individuals, but with a maximum of 50 g of carbohydrates per day) (116, 117).

The HPD involved a different macronutrient distribution, with a protein intake of 1.8 g/kg body weight per day, sourced from both animal and plant origins, amounting to approximately 136 g/day, with minor individual variations (70, 118, 119). The diet followed a non-calorically restricted model. A high protein intake combined with moderate fat intake was emphasized to distinguish it from a keto diet. Consequently, the macronutrient composition for each participant was as follows: 40% protein, 30% carbohydrates, and 30% fat (70).

The TRE involved the consumption of food without calorie counting, with meals monitored daily by a nutritionist via an online platform within a strictly defined eating window of 4–10 h, varying between individuals (80). The remaining 14–20 h were designated as a fasting period. During this fasting period, participants were advised to maintain adequate hydration.

### 2.5 Statistical analysis

In this study, a comprehensive set of statistical methods was employed to analyze the data collected from 150 overweight or obese female participants. Initially, the Shapiro–Wilk test was conducted to assess the normality of the numerical variables. Since all numerical variables exhibited non-Gaussian distribution, non-parametric methods were utilized for subsequent analyses. Numerical variables were summarized using medians, while categorical variables were presented as proportions. To evaluate the differences between diet groups, the Kruskal-Wallis test was applied. Following significant results from the Kruskal-Wallis test, the Dunn test was used for post-hoc analysis to identify specific group differences. To analyze repeated measurements within the same group over time, the Friedman test was employed. In addition to these methods, Linear Mixed-effects Models (LMM) were used to account for both fixed effects, such as diet and time, and random effects, such as individual differences among participants, being particularly valuable in handling repeated measures data, where multiple observations are made on the same subjects over time, allowing for the modeling of both within-subject and between-subject variability. In the LMM outputs, the residual variance (σ^2^) represents the unexplained variation after accounting for the fixed effects, while the random intercept variance (*τ*₀₀ID) captures the variability attributed to differences between subjects. The total number of observations (*N*) is indicated, and the marginal *R*^2^ provides a measure of the model’s explanatory power based on the fixed factors alone. These methods collectively provided a robust framework for analyzing the complex dataset, enabling the assessment of both between-group differences and within-subject changes over time. The data was collected, processed, and analyzed using R Core Team (2024). R: A language and environment for statistical computing. R Foundation for Statistical Computing, Vienna, Austria (120). A *p <* 0.05 was considered statistically significant, with a 95% confidence interval.

## 3 Results

### 3.1 Baseline characteristics and group comparability

Upon analyzing the numerical variables within the study, it was observed that the majority, with the exception of HDLc (*p* = 0.01), did not show significant differences across the six dietary groups. The results are detailed in Table 1. This consistency indicates that the participant populations were well-matched in terms of lipid profiles, kidney function, and glucose metabolism, thus minimizing the potential for these factors to confound the study results. The statistical difference in HDLc between the diet models suggests that the diets may have had distinct effects on this parameter. Post-hoc analysis using the Dunn test with Holm adjustment for *p*-values revealed statistically significant differences in HDLc between TRE and LCD (*p* = 0.05), LCD and VLED (*p* = 0.03), and LCD and HPD (*p* = 0.03). These findings imply that certain diets, particularly LCD, may have a less favorable impact on HDLc levels compared to others, such as TRE, VLED and HPD. The absence of significant variation in most other variables strengthens the internal validity of the study, indicating that observed differences in outcomes such as weight, BMI, or body composition can be more reliably attributed to the specific dietary interventions.

**Table 1:** Comparing numerical variables.

Variable	LED (*N* = 31)	TRE (*N* = 27)	KD (*N* = 23)	LCD (*N* = 26)	VLED (*N* = 20)	HPD (*N* = 23)	Kruskal–Wallis
LDLc	109.00	118.00	131.00	123.00	121.00	128.00	0.85
HDLc	53.00	54.00	48.00	46.50	54.50	55.00	**0.01**
Non-HDLc	123.00	138.00	155.00	147.50	135.00	150.00	0.92
Triglycerides	122.00	101.00	120.00	119.50	113.00	111.00	0.80
Creatinine	0.62	0.66	0.64	0.71	0.70	0.66	0.46
FBG	96	97	100	101	96	108	0.50

LDLc, Low-density lipoprotein cholesterol; HDLc, High-density lipoprotein cholesterol; FBG, Fasting blood glucose. Bold values indicate statistically significant results (*p* < 0.05).

Similarly, when analyzing the numerical variables of participants who followed the six types of diet therapy, no statistically significant differences were found, with the results presented in Table 2. The uniform distribution of these variables across the diet groups indicates that participants were well-matched in terms of demographic and lifestyle characteristics, thereby minimizing the potential for these factors to confound the study results. The lack of significant variation suggests that any observed differences in other outcomes, such as metabolic or physiological changes, can be more confidently attributed to the dietary interventions themselves, rather than underlying differences in participant characteristics. This consistency across categorical variables enhances the internal validity of the study and reinforces the reliability of the observed effects of the diets on the measured health outcomes.

**Table 2:** Comparing categorical variables.

Variable	Class	LED (*N* = 31)	TRE (*N* = 27)	KD (*N* = 23)	LCD (*N* = 26)	VLED (*N* = 20)	HPD (*N* = 23)	Pearson Chi^2^
Hyperuricemia	Yes	42%	41%	26%	54%	45%	35%	0.49
	No	58%	59%	74%	46%	55%	65%	
Lipid profile	Modified	35%	44%	52%	54%	50%	52%	0.74
	Not modified	65%	56%	48%	46%	50%	48%	
Vitamin D status	Optimal	32%	30%	13%	12%	25%	13%	0.51
	Insufficient	32%	26%	39%	38%	35%	52%	
	Deficit	36%	44%	48%	50%	40%	35%	
Insulin resistance	Yes	55%	56%	52%	69%	45%	70%	0.48
	No	45%	44%	48%	31%	55%	30%	
Diabetes status	Normal	53%	70%	48%	62%	75%	45%	0.55
	Prediabetes	27%	19%	30%	30%	15%	27%	
	Diabetes	20%	11%	22%	8%	10%	28%	
Education	≤9	6%	26%	13%	4%	5%	0%	0.11
	10–12	10%	15%	26%	38%	30%	22%	
	12–18	52%	33%	35%	38%	35%	52%	
	>18	32%	26%	26%	20%	30%	26%	
Age groups	18–24	10%	15%	17%	35%	30%	9%	0.40
	25–34	39%	33%	26%	27%	20%	30%	
	35–49	23%	33%	35%	8%	25%	30%	
	> = 50	29%	19%	22%	31%	25%	30%	
Marital status	Single	35%	41%	22%	31%	45%	26%	0.30
	Married	42%	44%	43%	50%	35%	26%	
	Divorced/Widowed	23%	15%	35%	19%	20%	48%	
Employment status	Housewife	19%	41%	35%	23%	35%	39%	0.29
	Student	16%	15%	13%	31%	30%	4%	
	Employee	42%	41%	43%	31%	20%	35%	
	Retired	23%	4%	9%	15%	15%	22%	
Menopause	Yes	29%	26%	22%	31%	30%	35%	0.95
	No	71%	74%	78%	69%	70%	65%	
Sleep deficit	Yes	45%	44%	35%	62%	50%	61%	0.39
	No	55%	56%	65%	38%	50%	39%	
Sedentary	Yes	52%	41%	35%	46%	65%	61%	0.30
	No	48%	59%	65%	54%	35%	39%	
Smoker	Yes	45%	67%	57%	46%	50%	39%	0.43
	No	55%	33%	43%	54%	50%	61%	

### 3.2 Anthropometric parameters analysis

#### 3.2.1 Body weight analysis

Within each diet group, the Friedman test revealed highly significant reductions in weight over time (*p <* 0.001 for all groups), indicating that all diets were effective in producing weight loss from the initial measurement to 6 weeks and from 6 weeks to 12 weeks. However, the Kruskal-Wallis test results indicated no statistically significant differences in weight among the diet groups at any of the time points (initial: *p* = 0.19, 6 weeks: *p* = 0.67, 12 weeks: *p* = 0.96), suggesting that the different diets did not produce significantly different weight outcomes when compared across groups. The results are presented in Supplementary Table 1.

The results of LMM indicated a significant weight reduction over time across all diets, with an average decrease of 5.29 kg at 6 weeks (95% CI: −6.80 to −3.78, *p <* 0.001) and 10.88 kg at 12 weeks (95% CI: −12.39 to −9.37, *p <* 0.001). However, the interaction between diet and time revealed that there were differences in weight loss among the studied diet categories. Participants who followed the TRE model experienced less weight reduction compared to those on the LED, both at the first follow-up (2.49 kg, 95% CI: 0.28 to 4.71, *p* = 0.028) and at the final follow-up (6.30 kg, 95% CI: 4.08 to 8.51, *p <* 0.001). Unexpectedly, similar results were observed with the VLED, showing less weight loss than LED at 12 weeks, with a positive estimate of (4.69 kg, 95% CI: 2.28 to 7.10, *p <* 0.001). Conversely, participants on isocaloric diets with varied macronutrient distributions—KD, LCD, and HPD—experienced significantly greater weight loss by the end of the study compared to those on LED, with negative estimates of −2.38 kg (95% CI: −4.69 to −0.06, *p* = 0.044), −3.01 kg (95% CI: −5.25 to −0.77, *p* = 0.009), and − 2.88 kg (95% CI: −5.20 to −0.56, *p* = 0.015), respectively. The results can be observed in Table 3.

**Table 3:** LMM analysis of weigh over time by diet group.

Predictors	Estimates	CI	*p*-value
T [6w]	−5.29	−6.80 to −3.78	<0.001
T [12w]	−10.88	−12.39 to −9.37	<0.001
Diet [TRE] × T [6w]	2.49	0.28–4.71	0.028
Diet [TRE] × T [12w]	6.30	4.08–8.51	<0.001
Diet [KD] × T [12w]	−2.38	−4.69 to −0.06	0.044
Diet [LCD] × T [12w]	−3.01	−5.25 to −0.77	0.009
Diet [VLED] × T [12w]	4.69	2.28–7.10	<0.001
Diet [HPD] × T [12w]	−2.88	−5.20 to −0.56	0.015
$\sigma^{2}$	9.14		
$\tau_{00}$ ID	278.15		
*N*	150		
Marginal *R*^2^	0.757		

T [6w], Time point 6 weeks; T [12w], Time point 12 weeks; CI, 95% Confidence interval.

#### 3.2.2 BMI analysis

The Kruskal-Wallis test indicated no statistically significant differences in BMI among the diet groups at the initial time point (*p* = 0.21), at 6 weeks (*p* = 0.91), and at 12 weeks (*p* = 0.65), suggesting that the changes in BMI were comparable across all diets throughout the study. However, the Friedman test revealed significant reductions in BMI within each diet group over time (*p <* 0.001 for all groups), indicating that each diet led to a significant decrease in BMI over the 12-week period. The results are presented in Supplementary Table 1.

The LMM analysis of BMI over time across different diets revealed significant reductions in BMI at both 6 and 12 weeks. Specifically, BMI decreased by an average of 1.94 kg/m^2^ at 6 weeks (95% CI: −2.50 to −1.37, *p <* 0.001) and by 3.83 kg/m^2^ at 12 weeks (95% CI: −4.39 to −3.26, *p <* 0.001) across all diets. Similar to the changes observed in body weight, the modification in BMI varied depending on the interaction between diet type and time. TRE was found to result in a smaller reduction in BMI at both the first follow-up (0.88 kg/m^2^, 95% CI: 0.05 to 1.72, *p* = 0.038) and at the end of the 12-week period (2.20 kg/m^2^, 95% CI: 1.37 to 3.04, *p <* 0.001) compared to LED. Similarly, and unexpectedly, VLED also resulted in a lesser reduction in BMI, but this was observed only at the end of the dietary program (1.67 kg/m^2^, 95% CI: 0.76 to 2.58, *p <* 0.001). Thus, both an isocaloric diet and one with significant caloric restriction showed reduced effectiveness in lowering or maintaining BMI over the long term. Conversely, LCD, an isocaloric diet that involved macronutrient redistribution, was associated in the LMM analysis with greater reductions in BMI at both 6 weeks (−1.00 kg/m^2^, 95% CI: −1.84 to −0.16, *p* = 0.020) and 12 weeks (−1.39 kg/m^2^, 95% CI: −2.23 to −0.54, *p* = 0.001), indicating its higher effectiveness in reducing BMI. The results are presented in Table 4. The model, with a marginal R^2^ value of 0.707, explained a substantial portion of the variance in BMI, while also highlighting significant individual differences in response to the diets.

**Table 4:** LMM analysis of BMI over time by diet group.

Predictors	Estimates	CI	*p*-value
T [6w]	−1.94	−2.50 to −1.37	<0.001
T [12w]	−3.83	−4.39 to −3.26	<0.001
Diet [TRE] × T [6w]	0.88	0.05–1.72	0.038
Diet [LCD] × T [6w]	−1.00	−1.84 to −0.16	0.020
Diet [TRE] × T [12w]	2.20	1.37–3.04	<0.001
Diet [LCD] × T [12w]	−1.39	−2.23 to −0.54	0.001
Diet [VLED] × T [12w]	1.67	0.76–2.58	<0.001
$\sigma^{2}$	1.30		
$\tau_{00}$ ID	22.60		
*N*	150		
Marginal *R*^2^	0.707		

T [6w], Time point 6 weeks; T [12w], Time point 12 weeks; CI, 95% Confidence interval.

#### 3.2.3 WC analysis

Over the course of the 12 weeks, significant reductions in WC values were observed across all diet categories, indicating that all diets were effective in reducing WC. However, the Kruskal-Wallis test revealed that there were no statistically significant differences in WC reductions between the different diet groups at any time point (initial: *p* = 0.15; 6 weeks: *p* = 0.75; 12 weeks: *p* = 0.93). The results are summarized in Supplementary Table 1.

The LMM analysis of WC over time revealed significant reductions in abdominal circumference across all diets, with an average decrease of 4.52 cm at 6 weeks (95% CI: −5.76 to −3.27, *p <* 0.001) and 8.74 cm at 12 weeks (95% CI: −9.99 to −7.49, *p <* 0.001). However, the interaction between diet and time showed varying effects. The KD was associated with an overall increase in abdominal circumference compared to LED, with an average increase of 9.00 cm (95% CI: 0.16 to 17.83, *p* = 0.046). Despite this, the KD led to significant reductions in abdominal circumference at both 6 weeks (−2.70 cm, 95% CI: −4.61 to −0.79, *p* = 0.006) and 12 weeks (−4.08 cm, 95% CI: −5.99 to −2.17, *p <* 0.001). Similarly, the LCD was effective in reducing abdominal circumference at 6 weeks (−2.10 cm, 95% CI: −3.95 to −0.25, *p* = 0.026) and 12 weeks (−4.03 cm, 95% CI: −5.87 to −2.18, *p <* 0.001). In contrast, TRE and VLED were associated with increases in abdominal circumference over time, particularly at 12 weeks (IF: 6.52 cm, 95% CI: 4.69 to 8.35, *p <* 0.001; VLED: 5.39 cm, 95% CI: 3.40 to 7.38, *p <* 0.001), suggesting they may be less effective for reducing abdominal circumference. The model explained 77.6% of the variance in abdominal circumference, underscoring the impact of both diet and time while also highlighting significant individual variability in response to these dietary interventions. These results are presented in Table 5.

**Table 5:** LMM analysis of abdominal circumference over time by diet group.

Predictors	Estimates	CI	*p*-value
Diet [KD]	9.00	0.16–17.83	0.046
T [6w]	−4.52	−5.76 to −3.27	<0.001
T [12w]	−8.74	−9.99 to −7.49	<0.001
Diet [TRE] × T [6w]	2.55	0.73–4.38	0.006
Diet [KD] × T [6w]	−2.70	−4.61 to −0.79	0.006
Diet [LCD] × T [6w]	−2.10	−3.95 to −0.25	0.026
Diet [VLED] × T [6w]	2.22	0.22–4.21	0.029
Diet [TRE] × T [12w]	6.52	4.69–8.35	<0.001
Diet [KD] × T [12w]	−4.08	−5.99 to −2.17	<0.001
Diet [LCD] × T [12w]	−4.03	−5.87 to −2.18	<0.001
Diet [VLED] × T [12w]	5.39	3.40–7.38	<0.001
$\sigma^{2}$	6.22		
$\tau_{00}$ ID	257.51		
*N*	150		
Marginal *R*^2^	0.776		

T [6w], Time point 6 weeks; T [12w], Time point 12 weeks; CI, 95% Confidence interval.

#### 3.2.4 WHR analysis

The analysis of WHR showed no statistically significant differences in this anthropometric parameter among the diet groups at the initial time point (*p* = 0.05) and at the 6-week mark (*p* = 0.33), suggesting that participants had comparable WHR across all diet groups in the early stages of the study. However, by 12 weeks, a significant difference in WHR was observed among the diet groups (*p* = 0.01), indicating that certain diets were more effective in reducing WHR over time. The Friedman test further confirmed significant reductions in WHR within each diet group over the 12-week period (*p <* 0.001 for all groups), demonstrating that all diets contributed to improvements in waist-to-hip ratios. The results are detailed in Supplementary Table 1.

The LMM analysis of WHR demonstrated that patients following isocaloric diets with varied macronutrient distributions, such as KD and LCD, were more effective in reducing WHR over the 12-week period compared to restrictive caloric diet. These reductions in WHR were statistically significant for both LCD: 0.38 units (95% CI: −0.70 to −0.06, *p* = 0.019) and KD: 0.36 units (95% CI: −0.69 to −0.03, *p* = 0.031). The model, which accounted for 3.6% of the variance in WHR, also indicated some individual variability in response to these dietary interventions. The results are detailed in Table 6.

**Table 6:** LMM analysis of WHR over time by diet group.

Predictors	Estimates	CI	*p*-value
T [6w]	0.25	0.03–0.46	0.026
Diet [KD] × T [6w]	−0.36	−0.69 to −0.03	0.031
Diet [LCD] × T [6w]	−0.38	−0.70 to −0.06	0.019
$\sigma^{2}$	0.19		
$\tau_{00}$ ID	0.03		
*N*	150		
Marginal *R*^2^	0.036		

T [6w], Time point 6 weeks; T [12w], Time point 12 weeks; CI, 95% Confidence interval.

### 3.3 Bioimpedance parameters analysis

#### 3.3.1 Fat mass analysis

**Figure 1:**
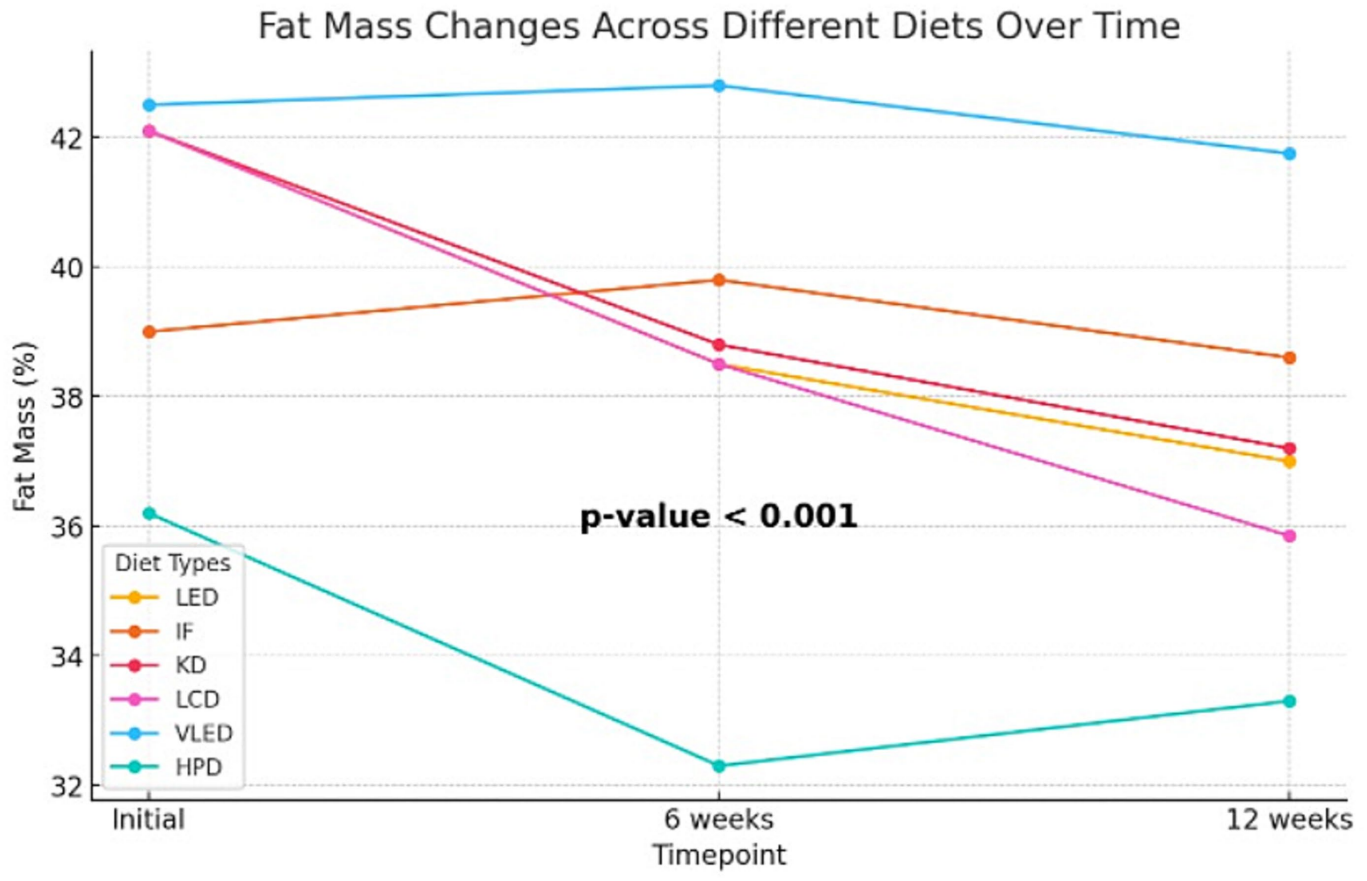
Fat mass and muscle mass changes across different diets over time.

Although the Kruskal-Wallis test did not identify statistically significant differences in body fat mass percentage after 6 weeks of diet therapy (*p* = 0.10), indicating that the reductions were comparable at this stage, by the end of the 12-week period, the impact of the diets on fat mass became more pronounced. Some diets led to greater reductions in body fat compared to others, with the differences reaching statistical significance (*p* = 0.002). Post-hoc analysis using the Dunn test revealed that the differences in fat mass at 12 weeks were statistically significant between HPD and TRE (*p* = 0.01) and between HPD and VLED (*p* = 0.003), highlighting that the HPD diet was more effective in reducing fat mass compared to TRE and VLED. The Friedman test revealed significant reductions in fat mass within each diet group over time (*p <* 0.001 for all groups except VLED, which had *p* = 0.04), confirming that all diets were effective in reducing fat mass. The slightly less significant result for the VLED group suggests that while fat mass decreased, the reduction was less pronounced compared to other diets. The results are presented in Supplementary Table 2 and Figure 1.

In addition to the Kruskal-Wallis and Friedman tests, the LMM was employed to analyze the effect of diet, time, and their interaction on fat mass, with diet and time as fixed effects and subject as a random effect. The analysis revealed that the HPD led to a significant reduction in fat mass, with an average decrease of 3.66% compared to LED (95% CI: −7.23 to −0.10, *p* = 0.044), indicating its effectiveness in fat mass reduction. Across all diets, fat mass decreased significantly over time, with reductions of 2.74% at 6 weeks (95% CI: −3.81 to −1.66, *p <* 0.001) and 4.72% at 12 weeks (95% CI: −5.79 to −3.64, *p <* 0.001), highlighting the substantial impact of time on fat mass loss. The interaction effects revealed that participants on the TRE model experienced less reduction in fat mass at both 6 weeks (1.78, 95% CI: 0.21 to 3.35, *p* = 0.027) and 12 weeks (3.40, 95% CI: 1.83 to 4.97, *p <* 0.001) compared to those on LED, suggesting that this type of nutritional intervention may be less effective over the long term. Conversely, the LCD was associated with a significant additional reduction in fat mass at 12 weeks (1.70, 95% CI: −3.29 to −0.11, *p* = 0.036), while the VLED showed less effectiveness in reducing fat mass, with a positive estimate of 3.62% (95% CI: 1.91 to 5.33, *p <* 0.001). The model, which explained approximately 60.1% of the variance in fat mass, also highlighted significant individual differences in how participants responded to the diets over time. Overall, the LMM results suggest that while time significantly contributes to fat mass reduction across all diets, isocaloric diets with specific macronutrient distribution such as HPD and LCD are more effective in achieving this reduction, whereas TRE and VLED may be less effective over the long term. The results are presented in Table 7.

**Table 7:** LMM analysis of fat mass over time by diet group.

Predictors	Estimates	CI	*p*-value
Diet [HPD]	−3.66	−7.23 to −0.10	0.044
T [6w]	−2.74	−3.81 to −1.66	< 0.001
T [12w]	−4.72	−5.79 to −3.64	< 0.001
Diet [TRE] × T [6w]	1.78	0.21–3.35	0.027
Diet [TRE] × T [12w]	3.40	1.83–4.97	< 0.001
Diet [LCD] × T [12w]	−1.70	−3.29 to −0.11	0.036
Diet [VLED] × T [12w]	3.62	1.91–5.33	< 0.001
$\sigma^{2}$	4.61		
$\tau_{00}$ ID	38.37		
*N*	150		
Marginal *R*^2^	0.601		

T [6w], Time point 6 weeks; T [12w], Time point 12 weeks; CI, 95% Confidence interval.

#### 3.3.2 FFM analysis

The changes in FFM over the 12-week period were comparable across the different diet categories studied, with no statistically significant differences at baseline (*p* = 0.30), at the intermediate time point (*p* = 0.10), or at the final assessment (*p* = 0.24). However, statistically significant differences were observed within each diet group (*p <* 0.001), except for the HPD group (*p* = 0.004). This suggests that, although all diets ultimately led to a reduction in FFM, HPD had a more favorable impact on preserving FFM, highlighting its potential as a more effective strategy for maintaining FFM compared to other diets in weight loss programs over time. The results are summarized in Supplementary Table 2.

The LMM analysis revealed that participants on the TRE model had a reduction in FFM of 15.81 kg (95% CI: −26.52 to −5.10, *p* = 0.004), those on the KD saw a decrease of 11.27 kg (95% CI: −22.47 to −0.08, *p* = 0.048), and participants on the LCD experienced a reduction of 13.61 kg (95% CI: −24.42 to −2.79, *p* = 0.014). The VLED was associated with a reduction of 16.67 kg (95% CI: −28.34 to −5.00, *p* = 0.005), and the HPD showed a decrease of 12.27 kg (95% CI: −22.03 to −3.51, *p* = 0.032). Additionally, there were significant reductions in FFM over time, with decreases of 12.77 kg at 6 weeks (95% CI: −22.03 to −3.51, *p* = 0.007) and 13.82 units at 12 kg (95% CI: −23.08 to −4.56, *p* = 0.004), irrespective of the diet followed. The model, which explained a modest portion of the variance in FFM (marginal R^2^ = 0.049), also highlighted individual differences in the response to the diets. These findings suggest that while all diets lead to a reduction in FFM, certain diets, particularly TRE and VLED, are associated with more pronounced decreases and the smallest reduction, which indicates greater long-term sustainability of the dietary program and underscores the importance of macronutrients regardless of caloric intake, was observed with KD. The results are presented in Table 8.

**Table 8:** LMM analysis of FFM over time by diet group.

Predictors	Estimates	CI	*p*-value
Diet [TRE]	−15.81	−26.52 to −5.10	0.004
Diet [KD]	−11.27	−22.47 to −0.08	0.048
Diet [LCD]	−13.61	−24.42 to −2.79	0.014
Diet [VLED]	−16.67	−28.34 to −5.00	0.005
Diet [HPD]	−12.27	−22.03 to −3.51	0.032
T [6w]	−12.77	−22.03 to −3.51	0.007
T [12w]	−13.82	−23.08 to −4.56	0.004
$\sigma^{2}$	342.94		
$\tau_{00}$ ID	80.72		
*N*	150		
Marginal *R*^2^	0.049		

T [6w], Time point 6 weeks; T [12w], Time point 12 weeks; CI, 95% Confidence interval.

#### 3.3.3 Trunk fat analysis

The percentage of trunk fat was reduced across all diet groups throughout the weight loss intervention, highlighting the effectiveness of both isocaloric diets with varied macronutrient distributions and calorically restrictive diets in reducing centrally located fat mass (*p <* 0.001 for all groups except VLED, which had *p* = 0.006). The results are detailed in Supplementary Table 2.

The LMM analysis of trunk fat over time across various diets demonstrated significant reductions in trunk fat at both the 6-week and 12-week marks, with an average decrease of 1.29% at 6 weeks (95% CI: −2.27 to −0.31, *p* = 0.010) and 3.14% at 12 weeks (95% CI: −4.12 to −2.16, *p <* 0.001) across all diets. However, the interaction between diet type and time indicated that the LCD was particularly effective, resulting in significant reductions in trunk fat at both 6 weeks (−2.36, 95% CI: −3.81 to −0.92, *p* = 0.001) and 12 weeks (−3.79, 95% CI: −5.24 to −2.34, *p <* 0.001). The KD also proved effective, with reductions observed at 6 weeks (−1.56, 95% CI: −3.06 to −0.06, *p* = 0.042) and 12 weeks (−2.03, 95% CI: −3.53 to −0.53, *p* = 0.008). In contrast, the TRE and VLED models were associated with smaller reductions in trunk fat, particularly at 12 weeks (TRE: 2.10, 95% CI: 0.67 to 3.53, *p* = 0.004; VLED: 2.57, 95% CI: 1.01 to 4.13, *p* = 0.001), suggesting they may be less effective for long-term trunk fat reduction. The HPD also contributed to trunk fat reduction at 12 weeks (−1.61, 95% CI: −3.11 to −0.12, *p* = 0.035). The model, which accounted for 54.8% of the variance in trunk fat, underscores the effectiveness of certain diets, particularly LCD and KD, while also highlighting significant individual variability in response to the dietary interventions. The results are detailed in Table 9.

**Table 9:** LMM analysis of trunk fat over time by diet group.

Predictors	Estimates	CI	*p*-value
Diet [LCD]	3.68	0.36–6.99	0.030
T [6w]	−1.29	−2.27 to −0.31	0.010
T [12w]	−3.14	−4.12 to −2.16	< 0.001
Diet [KD] × T [6w]	−1.56	−3.06 to −0.06	0.042
Diet [LCD] × T [6w]	−2.36	−3.81 to −0.92	0.001
Diet [TRE] × T [12w]	2.10	0.67–3.53	0.004
Diet [KD] × T [12w]	−2.03	−3.53 to −0.53	0.008
Diet [LCD] × T [12w]	−3.79	−5.24 to −2.34	< 0.001
Diet [VLED] × T [12w]	2.57	1.01–4.13	0.001
Diet [HPD] × T [12w]	−1.61	−3.11 to −0.12	0.035
$\sigma^{2}$	3.83		
$\tau_{00}$ ID	35.92		
*N*	150		
Marginal *R*^2^	0.548		

T [6w], Time point 6 weeks; T [12w], Time point 12 weeks; CI, 95% Confidence interval.

#### 3.3.4 Muscle mass analysis

Muscle mass analysis identified similar levels of lean mass percentage across all diets at both the initial and intermediate stages, with statistically significant differences emerging only at the conclusion of the 12-week nutritional intervention (*p* = 0.003). This indicates that certain diets demonstrated superior efficacy in increasing muscle mass percentage. While all diets promoted muscle mass growth, the TRE model was found to have a less pronounced effect on this BIA parameter (*p* = 0.03). Conversely, VLED did not show a statistically significant change in muscle mass (*p* = 0.16). The Friedman test confirmed significant increases in muscle mass within the LED, KD, LCD, and HPD groups (*p <* 0.001 for each), indicating that these diets were particularly effective in promoting muscle mass gains over the 12-week period. Post-hoc Dunn analysis further revealed statistically significant differences in muscle mass increases between the HPD and VLED groups (*p* = 0.001), as well as between the HPD and TRE groups (*p* = 0.03), highlighting that the HPD diet was more effective in increasing muscle mass compared to both VLED and TRE. These findings suggest that while most diets contributed to an increase in muscle mass, the extent of this increase varied, with certain diets, particularly HPD, LED, KD, and LCD, being more effective. The results are presented in Supplementary Table 2.

The LMM analysis of muscle mass over time across different diets revealed significant increases in muscle mass, with the HPD showing a particularly strong effect. HPD was associated with a significant overall increase in muscle mass compared to LED, with an average increase of 3.61% (95% CI: 0.12 to 7.11, *p* = 0.043). Additionally, the LCD demonstrated a notable increase in muscle mass at 6 weeks (1.81, 95% CI: 0.11 to 3.52, *p* = 0.037), while the KD was especially effective at 12 weeks (2.69, 95% CI: 0.92 to 4.45, *p* = 0.003). In contrast, VLED was associated with a decrease in muscle mass by 12 weeks (−2.02, 95% CI: −3.86 to −0.18, *p* = 0.031), indicating that it may not be as effective in preserving this parameter over time. The HPD diet continued to demonstrate strong effectiveness, with an additional increase in muscle mass at 12 weeks (2.95, 95% CI: 1.18 to 4.71, *p* = 0.001). The model accounted for 52.3% of the variance in muscle mass, underscoring the significance of diet choice in promoting muscle growth and the variability in individual responses. The results are presented in Table 10.

**Table 10:** LMM analysis of muscle mass over time by diet group.

Predictors	Estimates	CI	*p*-value
Diet [HPD]	3.61	0.12–7.11	0.043
T [6w]	1.47	0.32–2.62	0.012
T [12w]	2.73	1.58–3.88	< 0.001
Diet [LCD] × T [6w]	1.81	0.11–3.52	0.037
Diet [KD] × T [12w]	2.69	0.92–4.45	0.003
Diet [VLED] × T [12w]	−2.02	−3.86 to −0.18	0.031
Diet [HPD] × T [12w]	2.95	1.18–4.71	0.001
$\sigma^{2}$	5.31		
$\tau_{00}$ ID	35.90		
*N*	150		
Marginal *R*^2^	0.523		

T [6w], Time point 6 weeks; T [12w], Time point 12 weeks; CI, 95% Confidence interval.

#### 3.3.5 Body water analysis

At both baseline and at 6 weeks, no statistically significant differences were observed in the analysis of hydration status percentages between the diet groups; however, such differences emerged by the end of the study (*p* = 0.02). This finding suggests that there are similarities in the effectiveness of maintaining or even increasing body water percentage among the isocaloric diets with specific macronutrient distributions (KD, LCD, HPD) and the calorie-restricted diet (LED) (*p <* 0.001 for each). In contrast, the VLED group did not exhibit a statistically significant change (*p* = 0.07). Furthermore, post-hoc Dunn analysis revealed that the differences in body water percentage at 12 weeks were statistically significant between the HPD and VLED groups (*p* = 0.02), as well as between the HPD and TRE groups (*p* = 0.04), indicating that HPD was more effective in increasing body water compared to both VLED and IF. The results are detailed in Supplementary Table 2.

The LMM analysis revealed that HPD had a particularly strong effect, resulting in an average increase of 2.68% in body water compared to the LED (95% CI: 0.05 to 5.31, *p* = 0.046). Across all diets, body water increased significantly at both 6 weeks (1.51, 95% CI: 0.78 to 2.25, *p <* 0.001) and 12 weeks (4.03, 95% CI: 3.29 to 4.76, *p <* 0.001). However, the TRE and VLED models were associated with decreases in body water. The TRE diet showed reductions at both 6 weeks (−1.22, 95% CI: −2.30 to −0.14, *p* = 0.026) and 12 weeks (−3.36, 95% CI: −4.44 to −2.29, *p <* 0.001), while the VLED diet exhibited similar decreases at 6 weeks (−1.45, 95% CI: −2.62 to −0.28, *p* = 0.016) and 12 weeks (−3.02, 95% CI: −4.19 to −1.85, *p <* 0.001). In contrast, LCD was effective in increasing body water early on, with a significant rise at 6 weeks (1.27, 95% CI: 0.18 to 2.36, *p* = 0.022). The model accounted for 64.5% of the variance in body water, underscoring the significant impact of both diet and time on body water levels. The results are detailed in Table 11.

**Table 11:** LMM analysis of body water over time by diet group.

Predictors	Estimates	CI	*p*-value
Diet [HPD]	2.68	0.05–5.31	0.046
T [6w]	1.51	0.78–2.25	< 0.001
T [12w]	4.03	3.29–4.76	< 0.001
Diet [TRE] × T [6w]	−1.22	−2.30 to −0.14	0.026
Diet [LCD] × T [6w]	1.27	0.18–2.36	0.022
Diet [VLED] × T [6w]	−1.45	−2.62 to −0.28	0.016
Diet [TRE] × T [12w]	−3.36	−4.44 to −2.29	< 0.001
Diet [VLED] × T [12w]	−3.02	−4.19 to −1.85	< 0.001
$\sigma^{2}$	2.16		
$\tau_{00}$ ID	21.17		
*N*	150		
Marginal *R*^2^	0.645		

T [6w], Time point 6 weeks; T [12w], Time point 12 weeks; CI, 95% Confidence interval.

#### 3.3.6 BMR analysis

The analysis of BMR indicated no statistically significant differences in BMR among the diet groups at any of the measured time points (initial: *p* = 0.12; 6 weeks: *p* = 0.12; 12 weeks: *p* = 0.09), suggesting that participants maintained comparable BMR levels across all diets throughout the study. However, the Friedman test revealed highly significant reductions in BMR within each diet group over time (*p <* 0.001 for all groups), indicating that each diet contributed to a significant decrease in BMR over the 12-week period. The results are detailed in Supplementary Table 2.

The LMM analysis of BMR over time demonstrated significant reductions across all diets, with an average decrease of 56.87 kcal/day at 6 weeks (95% CI: −79.66 to −34.08, *p <* 0.001) and a more pronounced decrease of 105.81 kcal/day at 12 weeks (95% CI: −128.60 to −83.02, *p <* 0.001). However, the interaction between diet and time revealed that participants on the TRE diet experienced a smaller reduction in BMR at 12 weeks compared to those on the LED diet, with a positive estimate of 35.95 kcal/day (95% CI: 2.55 to 69.36, *p* = 0.035). This suggests that the TRE model may help preserve BMR to some extent over the longer term. Additionally, the analysis confirmed that caloric restriction leads to a reduction in BMR over time. The model also highlighted significant individual variability in BMR response, as evidenced by the large random effect variance, while the marginal R^2^ value of 0.069 indicates that the fixed effects explain 6.9% of the variance in BMR. The results are detailed in Table 12.

**Table 12:** LMM analysis of BMR over time by diet group.

Predictors	Estimates	CI	*p*-value
T [6w]	−56.87	−79.66 to −34.08	< 0.001
T [12w]	−105.81	−128.60 to −83.02	< 0.001
Diet [TRE] × T [12w]	35.95	2.55–69.36	0.035
$\sigma^{2}$	2078.06		
$\tau_{00}$ ID	99177.77		
*N*	150		
Marginal *R*^2^	0.069		

T [6w], Time point 6 weeks; T [12w], Time point 12 weeks; CI, 95% Confidence interval.

## 4 Discussion

Obesity, a critical public health issue with far-reaching implications, currently affects over 650 million adults globally. The prevalence of this condition has escalated dramatically over the past five decades, contributing to a substantial rise in associated comorbidities and healthcare burdens (121, 122). The wide range of dietary patterns, lifestyle behaviors, both psychological or emotional determinants and genetic factors all play a critical role in shaping an individual’s nutritional status, thereby influencing the distribution of adipose and lean tissue masses. These complex interactions are pivotal in determining body composition and significantly impact the efficacy of dietary strategies in managing body weight.

Prior to the implementation of any pharmacological or surgical interventions for obesity management, dietary therapy continues to serve as the foundational approach for long-term body weight regulation (121). Although numerous factors contribute to the interaction between diets and the weight loss process, the quantity and quality—particularly the macronutrient composition—of the daily consumed foods, alongside the timing of meals, are critical components in the effective management of excess body weight. These factors play a pivotal role in modulating metabolic responses and sustaining weight loss outcomes over time. The determination of optimal dietary strategies for effective weight management has been a subject of extensive debate and investigation among researchers, nutrition experts, and healthcare professionals, as well as within the general population (123, 124). Energy deficit is widely recognized as the fundamental determinant in the weight loss process (125). The U.S. Guidelines for the Management of Overweight and Obesity in Adults advocate for the regulation of daily caloric intake to a range of 1,200–1,500 kcal for women as a strategic measure for the prevention and treatment of obesity (126).

Dietary interventions have a significant impact on women with obesity, influencing various aspects of physical health, metabolic function, and psychological wellbeing. Calorie-restricted diets, particularly when combined with regular physical activity, have been shown to result in meaningful weight loss and improvements in metabolic markers such as insulin resistance, blood glucose control, and lipid profiles, which are crucial in reducing the risk of obesity-related diseases like type 2 diabetes and cardiovascular conditions (127). Different types of diets, such as LCDs, KDs, or Mediterranean have been compared, with evidence suggesting that Mediterranean diets, rich in healthy fats and fiber, tend to lead to more sustained weight loss and cardiovascular benefits, particularly in women (128, 129). However, while these dietary interventions can lead to physical health improvements, the psychological effects can be mixed. Many women with obesity experience improved self-esteem and reductions in symptoms of depression following weight loss, yet the rigid nature of certain diets may exacerbate issues related to body image, binge eating, and emotional stress, especially when weight loss is not sustained (130). Furthermore, research highlights that weight cycling, often triggered by repeated dieting attempts, can have detrimental effects on metabolism and emotional health, further complicating long-term weight management (131). Consequently, personalized dietary strategies that consider not only the metabolic but also the psychological needs of women with obesity are critical for achieving sustainable weight loss and improving overall wellbeing (132). However, the existing literature on the comparative impact of isocaloric diets with varying macronutrient distributions versus caloric restriction on bioelectrical impedance parameters in women with obesity or overweight remains limited.

LEDs have long been categorized into various subtypes based on the presence or absence of specific macronutrients balanced diets (commonly recommended by health professionals, consist of similar protein, carbohydrate, and fat ratios as nondieting populations, with the standard advice to “eat smaller portions” based on the rationale that simply reducing food intake is an effective treatment for obesity), one-food diets (rely on monotony and simplicity, where patients consume a single food at every meal, sometimes with minimal variation), elimination or reduction of one or more nutrients, vegetarian diets, formula diets, which can fall into various dietary categories, typically consist of a balanced mix of protein, carbohydrates, and low fat, and may be liquid or powder-based, often limiting intake to the formula alone or with minimal other foods, sometimes resembling a one-food diet and the last one, miscellaneous and magic diets, often fad-based and not fitting into standard categories, typically involve a combination of foods with questionable physiological rationale (133). Individuals with excess body weight are frequently advised to rigorously monitor their caloric intake at each meal, reinforcing the principle that effective weight reduction hinges on the balance between reduced energy consumption and increased physical activity. This strategy highlights the critical importance of creating and maintaining a negative energy balance, which is considered the cornerstone of sustainable weight management. The emphasis on “eat less, move more” remains a central doctrine in obesity treatment, supported by extensive evidence linking caloric restriction and enhanced physical exertion to successful long-term weight loss outcomes (134). According to Johnston et al. (135), caloric restriction was identified as the primary driver of weight loss, with macronutrient composition serving as a secondary factor. LED or diets with a caloric deficit ranging between 500 and 750 kcal have been recommended by various obesity societies (136–139). However, the proposed caloric deficit achieved through different nutritional interventions may be counteracted by physiological adaptations, which are based on the understanding that both energy intake and expenditure are dynamic processes (140). These adaptations can pose significant challenges to sustained weight loss, as the body may adjust to lower energy intake by reducing metabolic rate and altering energy expenditure (121).

However, beneficial effects on weight and body composition have been observed in association with caloric restriction, particularly in the reduction of fat mass, among women with obesity. These effects highlight the effectiveness of caloric restriction as a key strategy in decreasing body fat and improving overall body composition in this population (141). Similarly, findings from another study demonstrated that a daily caloric reduction of 500 kcal led to an average reduction in fat mass of up to 14% after 16 weeks of nutritional intervention (142). Despite these positive outcomes, LED has also been associated with undesirable effects on body composition, particularly a reduction in muscle tissue mass (143). Additionally, adherence to LED regimens has been shown to be challenging, often leading to increased appetite, which may further complicate the long-term sustainability of this dietary approach (144). In our research, the LED demonstrated favorable outcomes on anthropometric parameters, including body weight, BMI, WC, WHR, as well as on the reduction of total body fat mass and abdominal fat mass, with statistical significance at *p <* 0.001 for each parameter, as identified by BIA. Additionally, LED was found to promote an increase in muscle tissue mass by the end of the nutritional intervention (*p <* 0.001). However, subsequent comparative statistical analysis revealed that the long-term efficacy of LED was inferior to that of isocaloric diets with specific macronutrient distributions, such as the LCD, HPD, and KD. Moreover, a reduction in the BMR was observed among participants in the LED group, highlighting the impact of caloric restriction on basal metabolism and the earlier onset of a weight plateau compared to the other diets studied.

It has long been established that VLEDs are nutritional interventions where caloric intake does not exceed 800 kcal (145). Over the years, the definition of VLED has evolved concerning the degree of energy restriction. The implementation of fixed-energy VLEDs overlooks individual differences among obese individuals, making them physiologically unsupported (133). Therefore, calculating calorie intake based on kcal/kg of body weight following the determination of BMR would provide a more credible definition for these caloric restrictions, allowing for individualization based on both these characteristics and the degree of caloric restriction (133). VLEDs are designed to replace normal food consumption but should not be confused with meal replacement products such as shakes (28). Despite maintaining macronutrient distribution, even in cases of increased protein intake, VLEDs have demonstrated reductions in both muscle mass (by 25%) and fat mass (by 75%) (113). Significant weight loss has been observed without imbalances typically associated with hunger (113). However, concerns have been raised regarding the increased loss of muscle mass linked to the substantial energy deficit (146, 147). Regrettably, this dietary pattern is associated with several adverse effects, the most commonly observed being constipation, headaches, orthostatic hypotension, and hair loss, likely attributable to the resulting negative energy balance (148). Contrary to the favorable outcomes associated with the LED as demonstrated in our study, the results for the cohort adhering to the VLED did not exhibit similar efficacy. From a quantitative perspective on body mass reduction, the VLED intervention was markedly less effective. Specifically, when compared to other dietary regimens, including LED, the VLED group exhibited a lesser degree of weight reduction over the 12-week intervention period, culminating in a positive deviation of 4.69 kg, an indicator of suboptimal efficacy. This finding suggests that the long-term effectiveness of VLED is significantly diminished when juxtaposed with isocaloric diets that feature a targeted macronutrient distribution. This diminished efficacy in weight loss naturally extended to the BMI, where VLED was associated with a mere 1.67 kg/m^2^ reduction by the study’s conclusion. The inefficacy of VLED was further underscored by its impact on anthropometric measures; notably, contrary to expectations, participants in the VLED group experienced an increase in WC, with an average gain of 5.39 cm after 12 weeks—a concerning outcome in the context of abdominal adiposity. In terms of fat mass distribution, VLED was the only dietary intervention among those studied that resulted in comparatively modest reductions in total body fat percentage, achieving statistical significance at *p* = 0.04, in contrast to the more pronounced fat mass percentages reductions observed in isocaloric diets with macronutrient redistribution, which demonstrated significance at *p <* 0.001. Furthermore, interaction analyses between time and diet revealed that participants in the VLED group experienced a smaller decrement in adipose tissue both at the 6-week and 12-week marks, with a positive difference of 3.62%. A similar trend was observed in the reduction of centrally localized adipose tissue. Moreover, in evaluating the effects of VLED on muscle tissue percentage, it was the sole dietary regimen that did not elicit a statistically significant alteration in muscle mass over the 12-week intervention period (*p* = 0.16). This outcome suggests a lack of anabolic stimulus, which is typically expected with dietary interventions designed to preserve lean body mass. Additionally, these findings correspond with the observation of statistically insignificant changes in hydration status (*p* = 0.07) among participants in the VLED group. In summary, the data indicate that, in the long term, the VLED, characterized by significant caloric restriction, demonstrates a markedly lower efficacy in terms of both anthropometric parameters and BIA outcomes when compared to isocaloric diets with a balanced distribution of macronutrients. These results highlight the potential limitations of severe caloric restriction in achieving sustainable improvements in body composition and metabolic health.

The non-caloric restrictive nutritional interventions in our study that involved modifications in macronutrient distribution included the LCD, HPD, and KD. The outcomes associated with these diets are promising and significantly differ from those observed in caloric restrictive nutritional interventions.

LCDs are currently recommended as part of therapeutic strategies for various medical conditions, including obesity, diabetes mellitus, epilepsy, cardiovascular diseases, and even neoplasms (149–153). As a result, a diet can be classified as a LCD if it provides <45% of the total daily energy intake from carbohydrates (45). Within this category of diets, carbohydrate intake is reduced, while the amounts of protein and fat can vary (154). LCDs have been associated with several benefits related to weight reduction, which are attributed to decreased appetite, improved postprandial glycemic values, and enhanced insulin sensitivity (150, 155, 156). However, the actual carbohydrate intake within such dietary patterns may influence the extent of these observed benefits. Therefore, while LCDs can be effective in promoting weight loss, a severe reduction in carbohydrate intake, as seen in ketogenic diets (KDs), may negatively impact the lipid profile, particularly in individuals with diabetes mellitus (157, 158). Conversely, a randomized clinical trial demonstrated that both types of diets (LCD and KD) had comparable short-term effects in individuals with obesity and metabolic disorders (159). Furthermore, it has been observed that specific dietary patterns can alter body composition (160). However, Papadopoulou et al. (161) found no positive effects on sarcopenia. In our research, despite promoting weight loss at both intermediate and final assessments, it has been demonstrated that, in the long term, isocaloric diets are more effective for this anthropometric parameter compared to LED and VLED. Regarding BMI, participants following LCD exhibited significantly lower BMI values at both 6 and 12 weeks of nutritional intervention, indicating its superior efficacy in reducing this index over time. The LCD also proved effective in reducing other anthropometric parameters such as WC and WHR. Specifically, WC decreased by an average of 2.10 cm within the first 6 weeks, and by 4.03 cm by the end of the intervention. WHR values showed average reductions of 0.38 units at the intermediate assessment, with the most significant reduction occurring in the initial phase of the diet. The overall percentage of adipose tissue displayed statistically significant differences between the diet groups at the end of the nutritional interventions. Notably, compared to LED and VLED, LCD was associated with a significantly greater reduction in adipose tissue. Regarding the central distribution of adipose tissue, LCD demonstrated additional efficacy. The interaction between this nutritional model and time revealed that LCD led to significant reductions in trunk adipose tissue compared to LED, both at the intermediate and final assessments. Consequently, it can be stated that the long-term efficacy of maintaining this diet results in greater reductions in abdominal obesity, subsequently lowering the cardiometabolic risk associated with this type of obesity. In terms of FFM, a reduction was observed in this BIA parameter, although it was not significant compared to hypocaloric diets. However, LCD proved effective in increasing the percentage of muscle tissue, particularly at 6 weeks (*p <* 0.001), and the interaction with time demonstrated that LCD was more favorable than caloric restriction in promoting muscle tissue growth. Similarly, an improvement in hydration status was observed throughout the nutritional intervention period, with an increase of approximately 5.5%.

KD and HPD exert different effects on body composition due to their unique macronutrient compositions and metabolic impacts. KD, characterized by high fat intake and very low carbohydrate consumption, induces a metabolic state known as ketosis, where the body primarily uses fat as its energy source instead of glucose. This shift in metabolism leads to significant reductions in fat mass while helping to preserve lean mass due to the sparing effect of ketones on muscle protein catabolism (162). Additionally, KD has been shown to decrease overall body weight, with a greater proportion of weight loss attributed to fat reduction rather than muscle loss (163). However, the extremely low carbohydrate intake in KD may limit muscle growth, as carbohydrates play a role in muscle glycogen replenishment and protein synthesis (164). In contrast, HPD, which emphasizes a higher intake of protein while maintaining moderate carbohydrate and fat levels, are particularly effective in promoting muscle retention and even enhancing muscle hypertrophy during periods of caloric deficit (165). The high protein intake in HPD provides ample amino acids for muscle protein synthesis, reducing the likelihood of muscle loss during weight loss and improving muscle strength and function (166). HPD is also associated with greater satiety, which can aid in adherence to calorie-restricted diets, further enhancing body composition outcomes by reducing fat mass while preserving or even increasing lean mass (77). Compared to KD, HPD tends to result in a more favorable balance between fat loss and muscle preservation, particularly in physically active individuals (167). Therefore, while both diets can lead to improvements in body composition, HPDs offer a distinct advantage in preserving and promoting muscle mass, whereas KDs are more focused on reducing fat mass through metabolic adaptation (61, 168).

In present study, similar to the outcomes associated with the LCD intervention, another diet within the category of isocaloric diets with macronutrient distribution, the KD, demonstrated additional benefits for both anthropometric indices and parameters identified through BIA. Compared to caloric restriction, participants in the KD group experienced sustained weight loss. This nutritional intervention also proved effective in reducing WC, with a significant overall decrease in WC compared to the LED group at both 6 and 12 weeks. Regarding the interaction between diet and time, alongside LCD, KD also showed significant reductions in WHR during this period, with a reduction of 0.36 units at 6 weeks. The KD intervention also positively influenced BIA parameters, with the exception of BMR, where the reduction was similar to that observed in the LED group. Furthermore, KD was effective in reducing overall adipose tissue by approximately 5% by the end of the intervention. In conjunction with LCD, KD demonstrated efficacy in reducing central adiposity, particularly within the first 6 weeks (−1.56%), culminating in a percentage decrease of 2.03% by the end of the study. In terms of muscle tissue percentage, KD confirmed significant increases over the 12-week period (*p <* 0.001), with greater gains compared to caloric restrictive diets. This dietary model was the only one associated with the smallest reductions in FFM, showing a decrease of just 11.27 kg, which is a desirable outcome in all weight loss nutritional interventions. Alongside these results, KD also promoted a percentage increase in hydration status similar to LCD, by approximately 5%, but significantly higher compared to LED and VLED.

In our research, the HPD was also included among the isocaloric diets, and this intervention demonstrated long-term beneficial effects on the evaluated parameters. Regarding BIA, a higher protein intake over the 12-week period led to statistically significant differences in global fat mass compared to caloric restrictive diets. Furthermore, a substantial impact was observed in the reduction of adipose tissue predominantly located in the trunk region within this diet group, especially by the end of the study. Additionally, HPD proved to be the nutritional intervention with the most statistically significant impact on the percentage of muscle tissue compared to VLED, with an average increase of approximately 3.61% in the first 6 weeks compared to baseline. This strong effect on muscle mass growth was also superior to that observed with LED, further surpassing VLED. HPD continued to demonstrate robust efficacy, with an additional increase of 2.91% on average by 12 weeks, resulting in a total gain of approximately 6%—an impressive outcome for this BIA-evaluated parameter. While significant increases in hydration status were observed in both the LED and isocaloric diet groups, HPD exhibited an exceptionally strong effect, leading to an average increase of 2.68% compared to the hypocaloric diet.

TRE and other IF modalities exhibit distinct effects on body composition due to differences in their fasting and eating patterns. TRE involves restricting food intake to a specific period each day, typically 4–10 h, with the remaining hours of the day reserved for fasting (80). This method aligns food consumption with circadian rhythms, which has been shown to optimize metabolic health and reduce adiposity while preserving muscle mass. Patterson et al. (169) demonstrated in their study that TRE can lead to reductions in body fat and improvements in insulin sensitivity and lipid profiles, largely because it enhances metabolic efficiency without the extended fasting periods associated with more restrictive IF approaches. Conversely, other forms of intermittent fasting, such as alternate-day fasting (ADF) or the 5:2 diet, involve longer fasting intervals. ADF consists of alternating days of normal eating with days of significant caloric restriction or complete fasting, while the 5:2 diet involves eating normally for 5 days of the week and restricting caloric intake for the remaining 2 days (170). These extended fasting periods can lead to greater reductions in body weight and fat mass; however, they may also increase the risk of muscle loss if protein intake and resistance training are not adequately managed (170). The prolonged fasting associated with these methods can induce more pronounced metabolic stress and adaptations, potentially affecting muscle protein synthesis and overall muscle mass (171). TRE is often associated with fewer adverse effects compared to more extreme fasting regimens due to its shorter fasting periods, which help maintain consistent daily nutrient intake and support muscle preservation (87). Additionally, TRE has been linked to improvements in metabolic health markers such as reduced inflammation and enhanced glucose regulation, which contribute to favorable changes in body composition (172). In contrast, the more rigorous fasting schedules of ADF and the 5:2 diet can lead to greater fluctuations in metabolic rate and may require more careful monitoring to prevent potential muscle mass loss (167). Therefore, while both TRE and other IF strategies can be effective for weight loss, TRE offers a more sustainable approach to improving body composition with potentially fewer negative impacts on muscle mass and overall metabolic health. In our research, TRE was the only isocaloric diet without specific macronutrient distribution included in our study. This approach focused strictly on adhering to the recommended eating window, without additional qualitative or caloric calculations. Consequently, the results were as expected. Alongside VLED, TRE did not demonstrate long-term benefits for women with obesity and overweight. Compared to both LED and the isocaloric diets with macronutrient distribution, participants following TRE experienced less weight loss, which corresponded to a smaller reduction in BMI by the end of the 12-week nutritional intervention. Furthermore, increases in abdominal circumference were observed at both 6 and 12 weeks. TRE also failed to show benefits in parameters assessed through BIA, with statistical differences negatively favoring TRE in the long term for adipose tissue, muscle mass, and hydration status. Contrary to these unsatisfactory long-term weight loss results, the interaction between time and TRE indicated that participants in this category experienced a smaller reduction in BMR by the end of the study compared to the other diets, with a positive estimate of 35.95 kcal/day. This result suggests that TRE may help preserve BMR in the long term.

This study supports the hypothesis that isocaloric diets offer advantages in body weight, anthropometrics, and bioelectrical impedance markers in obese and overweight women. However, key limitations include the exclusive focus on females, limiting broader applicability, a small sample size reducing statistical power, and the 12-week duration, which may overlook long-term effects. These constraints call for cautious interpretation and highlight the need for further research with a larger, more diverse cohort over a longer period to confirm and expand these findings. A key limitation of this study is related to the cultural background of the population included in the sample. Certain dietary interventions, such as ketogenic-type diets (KTD), may present challenges in terms of adherence and applicability within specific cultural or regional contexts. Dietary preferences, traditional eating habits, and the availability of certain foods could significantly influence the feasibility and effectiveness of such interventions. These factors may introduce variability in compliance and outcomes, which should be carefully considered when interpreting the results. Future research should explore culturally tailored approaches to dietary interventions to enhance their applicability and effectiveness across diverse populations. On the other hand, another limitation of our research is the lack of data on participants’ menstrual cycles (presence, regularity, duration), which may influence metabolic and bioimpedance parameters. Future research should account for these variables to enhance result accuracy.

## 5 Conclusion

In conclusion, this study underscores the long-term advantages of isocaloric diets with specific macronutrient distributions, in optimizing body composition and anthropometric outcomes. These diets significantly outperformed caloric restrictive regimens, demonstrating superior reductions in BMI, WC, and total body adipose tissue, while concurrently promoting greater muscle mass retention and improved hydration status. Notably, the LCD and KD were particularly effective in reducing centrally located adipose tissue, a key factor in mitigating cardiometabolic risk. In contrast, the VLED and TRE interventions yielded suboptimal results, with participants experiencing less pronounced weight loss, minimal reductions in BMI, and, in the case of VLED, an unexpected increase in WC. Moreover, both VLED and TRE were associated with a less favorable impact on muscle mass and adipose tissue distribution, as assessed by BIA. The diminished efficacy of VLED coupled with the observed reduction in basal metabolic rate in TRE, highlights the potential drawbacks of severe caloric restriction and time-restricted eating in achieving sustainable improvements in body composition and metabolic health. These findings suggest that isocaloric diets with targeted macronutrient distribution may offer a more effective and sustainable approach to weight management and metabolic health in individuals with obesity and overweight.

## Data Availability

The raw data supporting the conclusions of this article will be made available by the authors, without undue reservation.
